# Supplementary material for: Milk Protein Adsorption on Metallic Iron Surfaces
Source: Nanomaterials (Basel). 2023 Jun 14;13(12):1857. doi: 10.3390/nano13121857 (PMC10304772; doi:10.3390/nano13121857)
Supplement: Supplementary file 1 [file nanomaterials-13-01857-s001.zip › Supporting-information.pdf]

Supporting Information

Milk Protein Adsorption on Metallic Iron Surfaces

Parinaz Mosaddeghi Amini, Julia Subbotina, Vladimir Lobaskin

School of Physics, University College Dublin, Belfield, Dublin 4, Ireland

Contents

1 Free energies of adsorption for 22 amino acid side chains on zero-valent FeNPs 2

2 RMSD plot for milk proteins equilibration with MD calculations 3

3 Example of FeNP size-dependent interaction of α lactalbumin 4

4 Comparison of interaction of the selected milk proteins with zero-valent FeNPs 5

4.1 αs1casein 5
4.2 αs2 casein 6
4.3 β casein 7
4.4 α lactalbumin 8
4.5 β lactoglobulin 9
4.6 Bovine Serum Albumin 10

5 FeNP validation with experimental approach: Hen egg white lysozyme binding properties on zero-valent FeNP 11

1 Free energies of adsorption for 22 amino acid side chains on zero-valent FeNPs

Table S1: Adsorption free energies (in kJ/mol) for side chain analogues (SCA) on the Fe-100, Fe-110 and Fe-111 surfaces from Adaptive Well-Tempered Metadynamics (AWT-MetaD) simulations (PMF minimum).

SCA	class^a	%^{b}	$T-group^c$	Charge	E_{min}Fe-100	E_{min}Fe-110	E_{min}Fe-111
ALA	H	7.5	CH_3	0	-0.87	2.86	-2.86
ARG	C^+	5.2	NH_2	+1	-74.80	-62.03	-75.68
ASP	C^-	5.2	O	−1	-35.35	-36.90	-35.02
ASN	P	4.6	NH_2	0	-38.87	-35.62	-41.47
CYS	P	1.8	CH	0	-16.54	-21.79	-21.02
GAN	P	6.3	COOH	0	-54.42	-38.56	-57.18
GLN	P	4.1	NH_2	0	-46.50	-36.98	-48.26
GLU	C^-	6.3	O	−1	-37.12	-38.67	-43.09
GLY	P	7.1	CH_2	0	-52.90	-48.26	-54.15
HID	A	2.2	NH	0	-54.79	-52.62	-62.75
HIE	A	2.2	NH	0	-56.98	-49.36	-67.02
ILE	H	5.5	CH_3	0	-29.97	-32.79	-28.53
LEU	H	9.1	CH_3	0	-1.58	-21.70	-20.30
LYS	C^+	5.8	NH_2	+1	-42.42	-36.93	-37.98
MET	H	2.8	NH_2	0	-35.34	-33.95	-37.89
PHE	A	3.9	CH	0	-49.63	-35.93	-62.52
PRO	P	5.1	CH_2	0	-91.29	-71.25	-78.28
SER	P	7.4	OH	0	-16.41	-17.32	-17.86
THR	P	6.0	OH	0	-25.40	-25.25	-25.05
TRP	A	1.3	NH	0	-80.59	-48.05	-98.49
TYR	A	3.3	OH	0	-67.65	-43.34	-79.96
VAL	H	6.5	CH_3	0	-19.68	-16.41	-17.00

^{a} H = hydrophobic, P = polar, A = aromatic, and C = charged, ^{b}the percentage of amino acids composition in proteins ([3]), ^{c} Terminating group in SCA. Histidine exists in two forms (HID) and (HIE) depending on the location of the protonation on the nitrogen atom in the imidazole ring of its side chain. In histidine D (HID), the proton is on the δ-nitrogen, and in histidine E (HIE), the proton is on the ϵ-nitrogen. GAN is the neutral form of the negatively charged Glutamic acid (GLU). The negatively charged form is due to the presence of a carboxyl group in its side chain, which can donate a hydrogen ion to a water molecule to form a hydroxide ion and a negatively charged carboxyl group. In neutral form the side chain carboxyl group has been modified so that it has a neutral charge at physiological pH.

2 RMSD plot for milk proteins equilibration with MD calculations

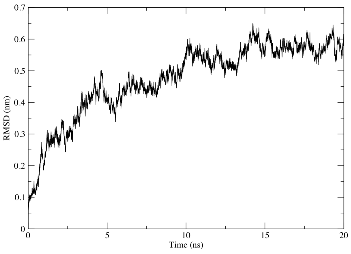

(a) AS1C

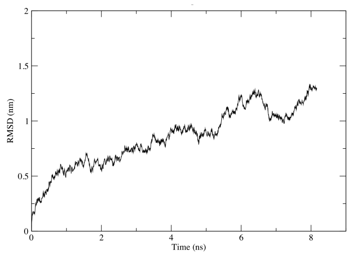

(b) AS2C

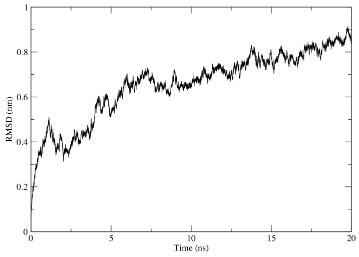

(c) BC

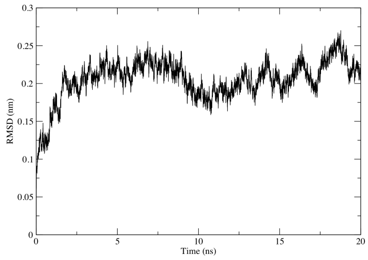

(d) ALAC

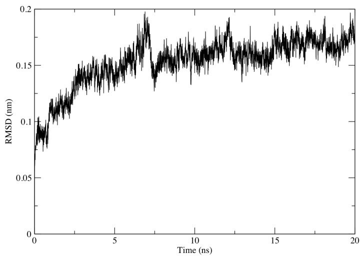

(e) BLAC

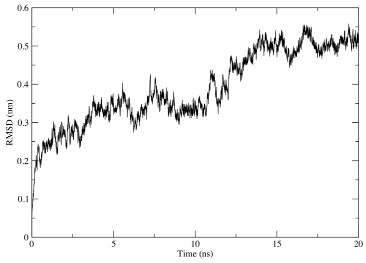

(f) BSA

Figure S1: Time series plots for 6 selected milk proteins: (a) AS1C, (b) AS2C, (c) BC, (d)BLAC, (e) ALAC and (f) BSA on FeNP shows the RMSD levels off to between $\sim$ 0.2 - 0.7 nm for 20 ns shows that the structure is very stable. We used the trajectory to process the diffusion of the protein during equilibration inside the cell.

3 Example of FeNP size-dependent interaction of α lactalbumin

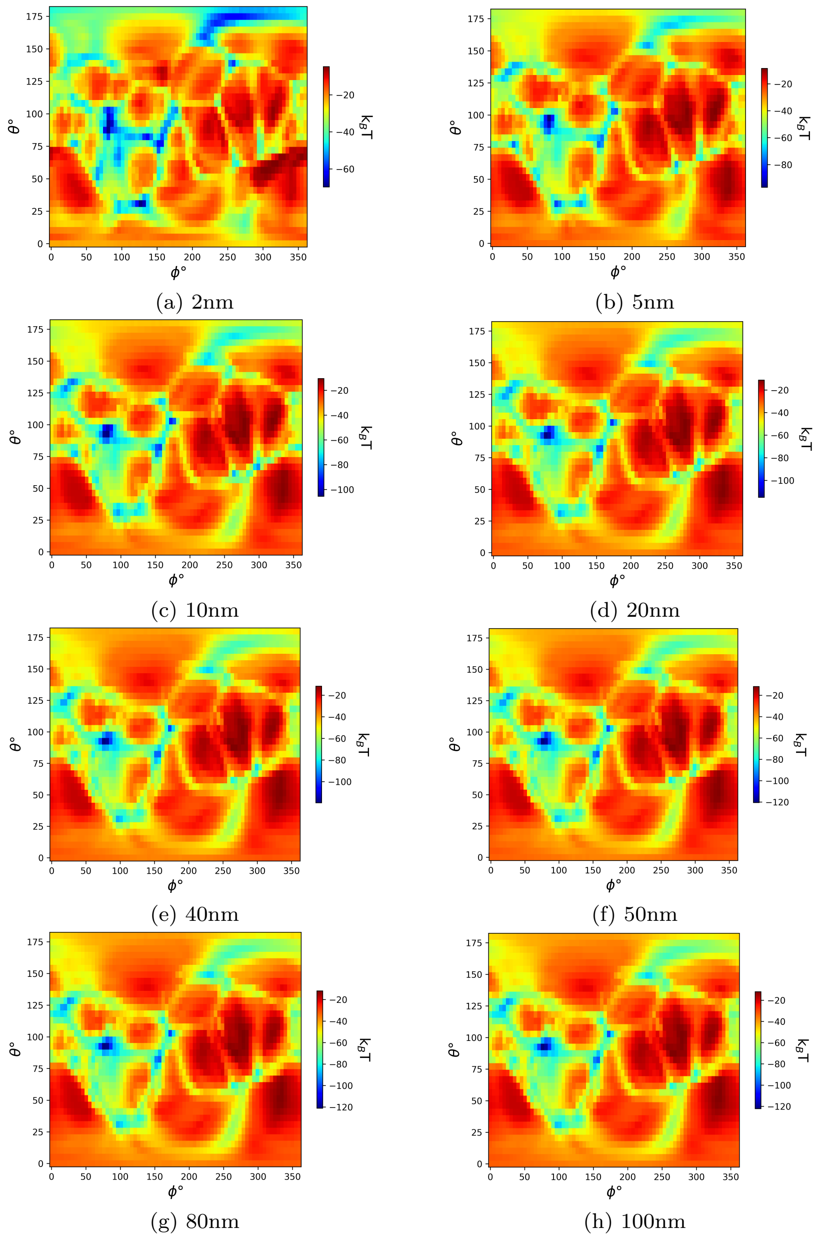

(a) 2nm (b) 5nm (c) 10nm (d) 20nm (e) 40nm (f) 50nm (g) 80nm (h) 100nm

Figure S2: The heatmap evolution based on the size of the FeNP-110 for α lactalbumin.

4 Comparison of interaction of the selected milk proteins with zero-valent FeNPs

4.1 αs1 casein

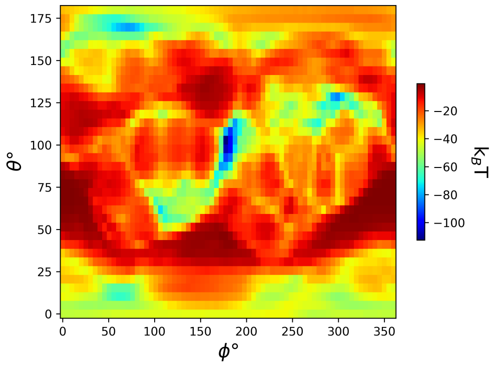

(a) Fe-100

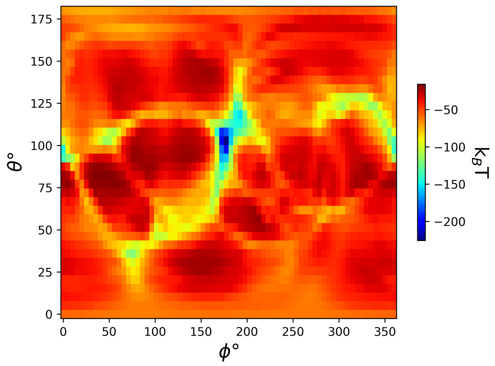

(b) Fe-110

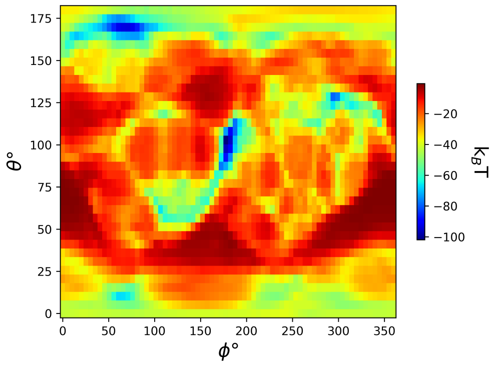

(c) Fe-111

Figure S3: Heatmap for αs1 casein on (a) Fe-100, (b) Fe-110, (c) Fe-111

4.2 αs2 casein

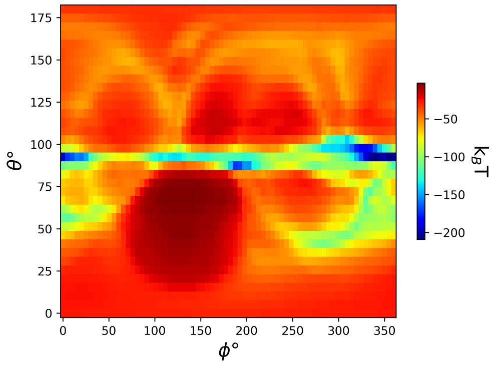

(a) Fe-100

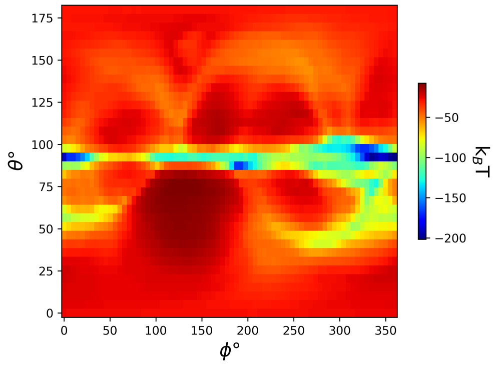

(b) Fe-110

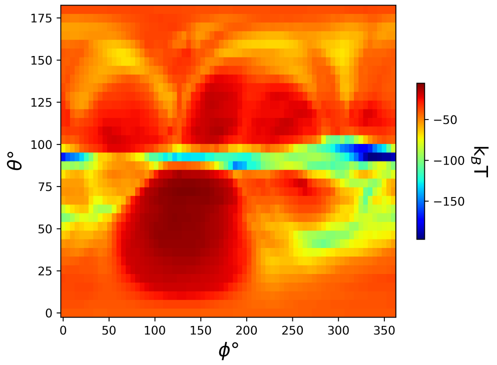

(c) Fe-111

Figure S4: Heatmap for αs2 casein on (a) Fe-100, (b) Fe-110, (c) Fe-111

4.3 β casein

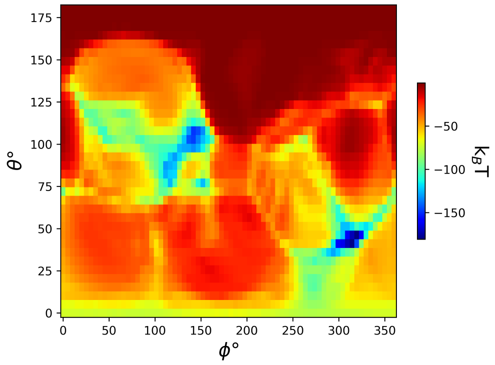

(a) Fe-100

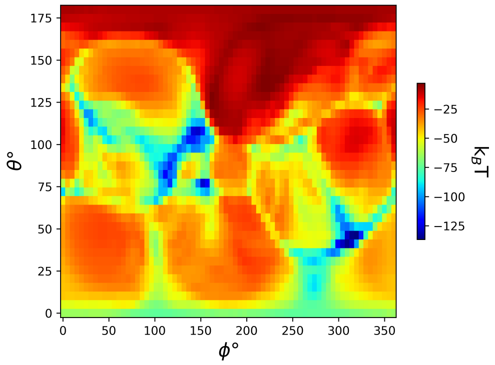

(b) Fe-110

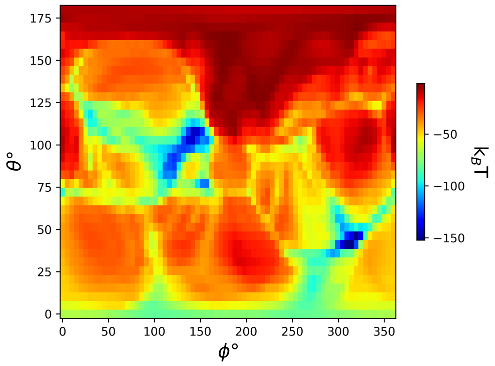

(c) Fe-111

Figure S5: Heatmap for β casein on (a) Fe-100, (b) Fe-110, (c) Fe-111

4.4 α lactalbumin

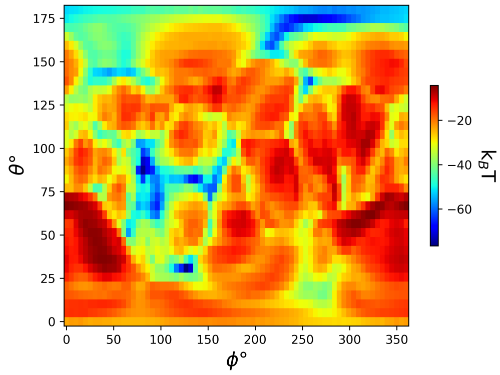

(a) Fe-100

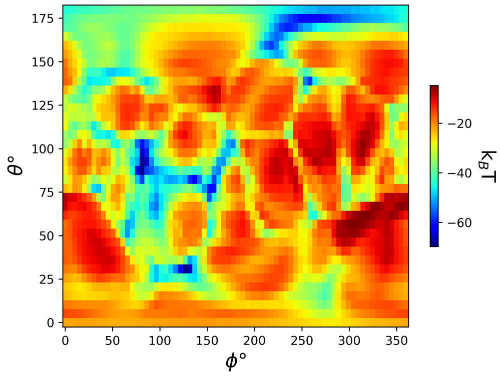

(b) Fe-110

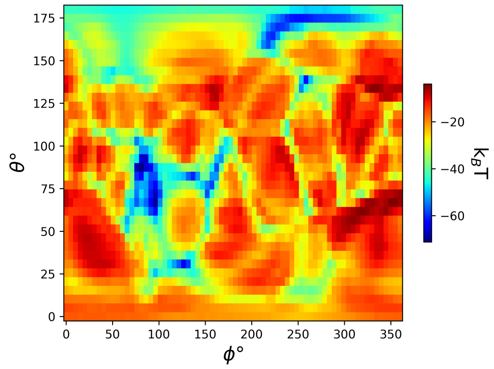

(c) Fe-111

Figure S6: Heatmap for α lactalbumin on (a) Fe-100, (b) Fe-110, (c) Fe-111

4.5 β lactoglobulin

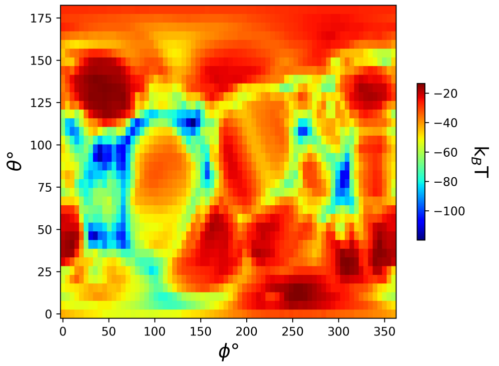

(a) Fe-100

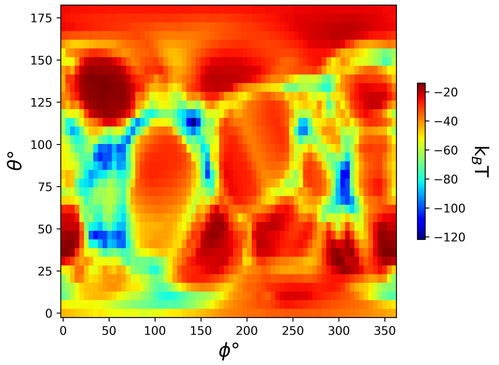

(b) Fe-110

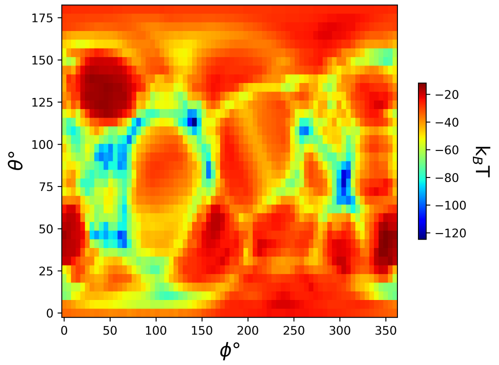

(c) Fe-111

Figure S7: Heatmap for β lactoglobulin on (a) Fe-100, (b) Fe-110, (c) Fe-111

4.6 Bovine Serum Albumin

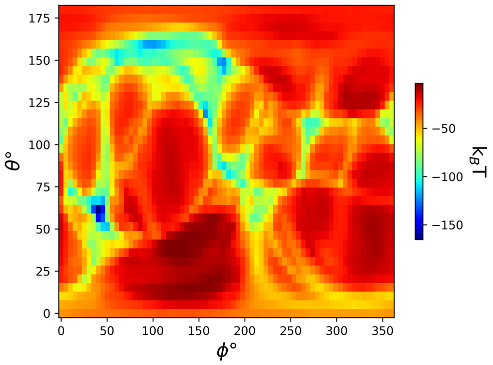

(a) Fe-100

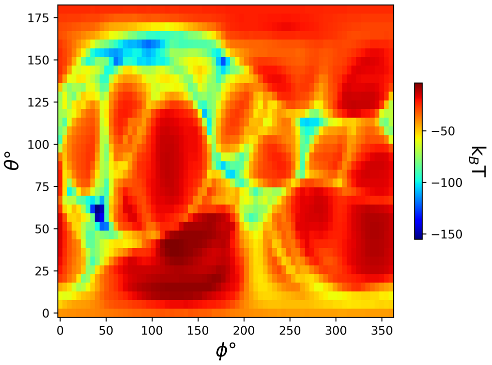

(b) Fe-110

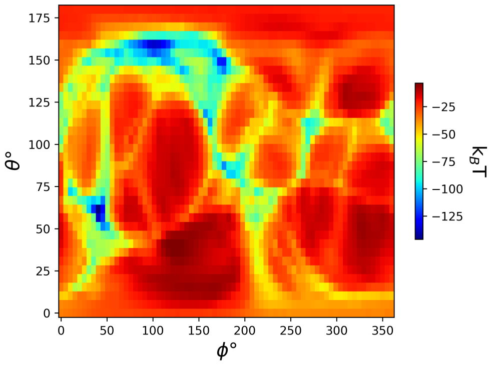

(c) Fe-111

Figure S8: Heatmap for Bovine Serum Albumin on (a) Fe-100, (b) Fe-110, (c) Fe-111

5 FeNP validation with experimental approach: Hen egg white lysozyme binding properties on zero-valent FeNP

Interaction of zero-valent iron NP with proteins has been studied with different experimental methods [1, 2]. Here we compare our results with the available biophysical and Molecular Docking studies results for hen egg white lysozyme (HEWL). The study examined the way in which HEWL interacts with zero-valent FeNPs (35-45 nm) using spectroscopic and docking analyses. The analysis of zeta potential indicated that introducing FeNP to HEWL can result in a wider distribution of charges in the protein-NP system. Furthermore, a dynamic light scattering study showed that potential for suspension in the protein-NP system increases by addition of FeNPs to HEWL. Fluorescence quenching studies also revealed that hydrogen bonds and van der Waals interactions contribute to the protein-NP system and that static and dynamic quenching mechanisms occur. The fluorescence data showed a significant exposure of hydrophobic residues to the solvent. After conducting experimental measurements, computational docking studies were utilized to further investigate the interactions between Fe NP and HEWL (PDB ID: 6lys) and to identify the specific amino acids involved in site-specific binding. The molecular docking was carried out in the presence of a water-coated NPs, and the resulting binding energies were determined to be -230.92 kJ/mol, indicating a strong binding affinity between the decorated NPs and the protein [1]. Taking the same protein PDB structure and temperature and size 35 nm and 45 nm for FeNP-110 in *UA* model we measured the averaged minimum free energy of adsorption equal to -407.74 KJ/mol at $\theta = 90$ and $\phi = 0$. The study also shows that by increasing temperature the interaction between HEWL and FeNP reduces. Based on their result at 298 K $\Delta G = -6.82$ (KJ/mol), $\Delta H = -90.90$ (KJ/mol) and $\Delta S = -298.11$ (J/mol K) that are Gibbs free energy, enthalpy and entropy difference. Figure S9 shows the interaction of the HEWL with FeNP and the nearest amino acids to the surface.

References

[1] Zahra Aghili et al. “Investigating the interaction of Fe nanoparticles with lysozyme by biophysical and molecular docking studies”. In: *PloS one* 11.10 (2016), e0164878.

[2] Tabassom Sedaghat Anbouhi et al. “Albumin binding, anticancer and antibacterial properties of synthesized zero valent iron nanoparticles”. In: *International journal of nanomedicine* 14 (2019), p. 243.

[3] Georges Trinquier and Yves-Henri Sanejouand. “Which effective property of amino acids is best preserved by the genetic code?” In: *Protein engineering* 11.3 (1998), pp. 153–169.

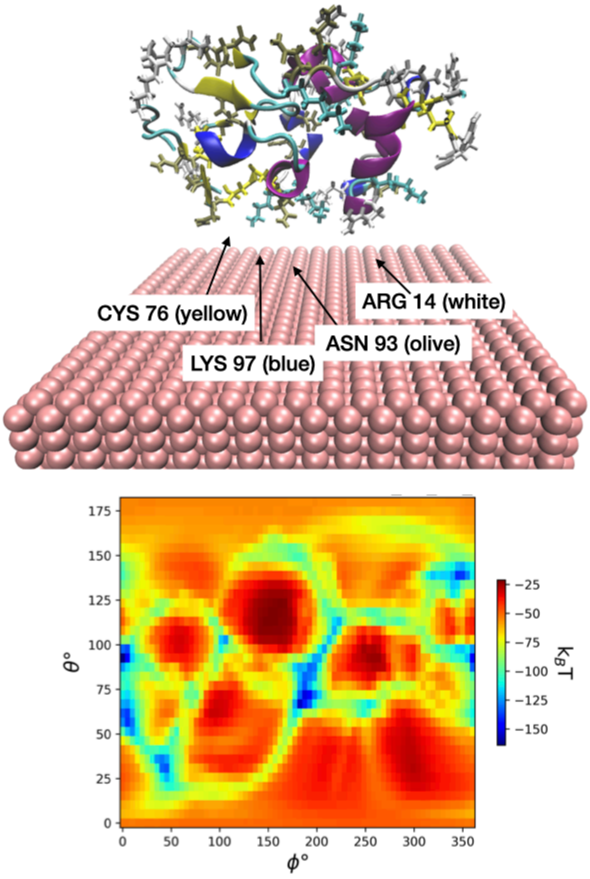

Figure S9: adsorption on HEWL on Fe-110 using *UA* and VMD. The figure shows the nearest amino acids of the protein to the slab (LYS, ASN, CYS, and ARG).
